# Exploration of cotton leaf curl virus resistance genes and their screening in *Gossypium arboreum* by targeting resistance gene analogues

**DOI:** 10.1093/aobpla/ply067

**Published:** 2018-10-16

**Authors:** Rakhshanda Mushtaq, Khurram Shahzad, Shahid Mansoor, Zahid Hussain Shah, Hameed Alsamadany, Tahir Mujtaba, Yahya Al-Zahrani, Hind A S Alzahrani, Zaheer Ahmed, Aftab Bashir

**Affiliations:** 1 Agriculture Biotechnology Division, National Institute for Biotechnology and Genetic Engineering, Faisalabad, Pakistan; 2 Department of Biotechnology, Pakistan Institute of Engineering and Applied Sciences, Nilore, Islamabad, Pakistan; 3 Department of Plant Breeding and Genetics, Faculty of Basic and Applied Sciences, University of Haripur, Haripur, Khyber Pakhtunkhwa, Pakistan; 4 Department of Plant Breeding and Genetics, Pir Mehr Ali Shah Arid Agriculture University, Rawalpindi, Pakistan; 5 Department of Biological Sciences, King Abdulaziz University, Jeddah, Saudi Arabia; 6 Plant and Forest Biotechnology Umea, Plant Science Centre (UPSC), Swedish University of 12 Agriculture Sciences (SLU), Umea, Sweden; 7 College of Science, Imam Abdulrahman Bin Faisal University, Dammam, Saudi Arabia; 8 Department of Plant Breeding and Genetics, University of Agriculture Faisalabad, Faisalabad, Pakistan; 9 Faculty of Biological Sciences, Forman Christian College University, Lahore, Pakistan

**Keywords:** Asymptomatic, CottonGen, ESTs, expression, motifs, symptomatic

## Abstract

Cotton leaf curl virus (CLCuV) disease is one of the major limiting factors in cotton production, particularly in widely cultivated *Gossypium hirsutum* varieties that are susceptible to attack by this virus. Several approaches have been employed to explore putative resistance genes in another cotton species, *G. arboreum*. However, the exact mechanisms conferring disease resistance in cotton are still unknown. In the current study, we used various approaches to identify possible resistance genes against CLCuV infection. We report the identification and isolation of a set of genes involved in the resistance response to viral infestation. PCR products containing genomic DNA gave multiple amplifications with a single primer in most reactions, and 38 fragments were cloned from *G. arboreum* and *G. hirsutum*. The sequences of cloned fragments belonged to various pathway genes and uncharacterized proteins. However, five amplified fragments (RM1, RM6, RM8, RM12 and RM31) showed similarity with R genes. Maximum homology (94 %) was observed with *G. raimondii* toll/interleukin receptor-like protein. BLAST search showed the homology of all resistance gene analogues (RGAs) with more than one chromosome, and multiple hits were observed on each chromosome for each RGA. Expression analysis through RT–PCR identified variable expression levels of the different RGAs in all tested genotypes. The expression level of RGAs differed between symptomatic and asymptomatic plants, with the exception of RGA 395, whose expression level was the same in both diseased and healthy plants. Knowledge of the interaction of these genes with various cotton pathogens could be utilized to improve the resistance of susceptible *G. hirsutum* and other plant species.

## Teaser

The cotton species, *Gossypium hirsutum*, is sensitive to attack by cotton leaf curl virus. The devastating virus attacks the cotton plant when it gets a chance to get into the system, consequently resulting in curling of the leaves. Whitefly serves as a viral vector, and when feeding on the cotton plant, break the protective barrier and hence result in cotton infestation. Another species of cotton, *Gossypium arboreum*, is totally resistant to this viral disease. Whitefly also feed on *G. arboreum* and transmit virus, but this species has a natural defence system that does not allow the virus to cause infection and disease. The present study identified some genes of the defence system of *G. arboreum* that might contribute to make this species resistant to leaf curl disease in cotton.

## Introduction

Analogous to the vertebrate immune system, the resistance (R) genes in plants recognize pathogen effectors (avirulence factors) and awaken the defence system of plants to combat attack (Ellis and Jones 1998; Chen *et al.* 2015). Many R genes have been isolated (Zhang and Zhou 2010), characterized and used in crop improvement programmes with varying degrees of success (Gururani *et al.* 2012). R genes are race specific and exhibit resistance only in the presence of cognate pathogen effectors. Resistance is often manifested as a hypersensitive response at the site of invasion, which restricts pathogen entry (Heath 2000).

Five different classes of R genes are known (Chen *et al.* 2015). To date the largest class of R genes are nucleotide-binding site leucine-rich repeats (NBS-LRR) (Dangl and Jones 2001; Chen *et al.* 2015). *Arabidopsis thaliana* is reported to contain 149 NBS-LRR (Meyers 2003; Khan *et al.* 2016), and 653 genes of this class have been reported in *Oryza sativa* (Shang *et al.* 2009). TIR-NBS-LRR and CC-NBS-LRR are two subclasses of NBS-LRR that contain N terminal toll/interleukin-1 receptor (TIR) and coiled coil domains (CC), respectively (DeYoung and Innes 2006). Other classes of R genes produce surface-localized pattern recognition receptors (PRR). These include receptor-like kinases and receptor-like proteins (Monaghan and Zipfel 2012). Sixty-three resistance gene analogue (RGA) clusters have been reported in the diploid D-genome of *G. raimondii*, while Wang *et al.* (2013) and Wei *et al.* (2013) identified 355 NBS-encoding genes in the *raimondii* genome.

Expressed sequence tags (ESTs) are an important tool for gene discovery (Hughes and Friedman 2005), molecular marker identification (Michalek *et al.* 2002), microarray development (Alba *et al.* 2004) and comparative genomics (Schlueter *et al.* 2004). Expressed sequence tags can be assigned a known function on the basis of homology with known genes found through BLAST search. Expressed sequence tag homologues of R genes can be used to design markers linked to functional R genes (Brugmans *et al.* 2008). Various types of resistance-gene-based markers include resistance gene analogue polymorphism (RGAP), NBS profiling and inter small RNA polymorphism (iSNAP) (Poczai *et al.* 2013). Markers based on ESTs have genic function and show linkage to transcriptional regions (Bozhko *et al.* 2003). Expressed sequence tag- and RGA-based markers have also been used in cotton (Chee *et al.* 2004; Buyyarapu *et al.* 2011). Cotton leaf curl is a disease of viral origin, transmitted by the whitefly *Bemisia tabaci.* This disease is difficult to control owing to the occurrence of several virulent viral strains or related species (Rahman *et al.* 2017).

In this study, we used RGA and EST homologues of R genes to reveal their expression pattern in cotton leaf curl virus (CLCuV)-resistant and -susceptible cotton genotypes. The RGA and EST homologues expressed in resistant genotypes can be further studied to find genes involved in CLCuV resistance. The aim of the current study was to explore the disease resistance genes in the published literature/GenBank and to screen for them in *G. arboreum* using degenerate primers on genomic DNA and RT–PCR on RGAs detected from ESTs.

## Materials and Methods

### Degenerate primer design

R genes of different classes were searched in cotton species at the NCBI. The nucleotide sequences of R genes were aligned using the multiple sequence alignment tool in the CLCBIO workbench. The conserved regions of alignments were used to design degenerate primers. Primer length was restricted to 24 mer. A maximum of four degeneracies was allowed per primer. Degeneracy was not allowed at the 3′ end of the primer. The primer sequences are shown in **Supporting Information—Table S1**.

### PCR on genomic DNA of cotton

Six cotton genotypes (one *G. arboreum* (Ravi) and five *G. hirsutum* (NIBGE-2, NIBGE-115, N-253, IR-3701 and Coker)) were used for genomic DNA isolation from young cotton leaves following the CTAB (cetyl trimethylammonium bromide) method. Isolated DNA was quantified using an ultra-spectrophotometer. The PCR recipe was as follows: 5 µL genomic DNA (50 ng µL^−1^), 5 µL 10× *Taq* buffer, 4 µL of 25 mM MgCl_2_, 1 µL of 10 mM dNTPs, 1 µL of each forward and reverse primer (50 ng µL^−1^), 1 µL of *Taq* DNA polymerase (2 units per µL) and PCR water to make a final volume of 50 µL.

### Cloning of PCR fragments in TA vector

All the PCR fragments amplified in CLCuV-resistant *G. arboreum* and the fragments differentially expressed in tolerant *G. hirsutum* genotypes were cloned in TA vector. For this purpose the PCR bands were gel eluted and ligated in TA vector (pTZ57R/T; Thermo Fisher Scientific, USA). The ligation was used to transform *Escherichia coli* competent cells (DH5∞). The transformed cells were spread on Luria-Bertani (LB) agar plates and the white colonies were cultured to isolate plasmid. The presence of clones was confirmed by restriction analysis using XbaI, SmaI, HindIII, EcoRI, BamHI, PstI, EcoRV and SacI.

### Analysis of sequencing data

Ninety-eight cloned fragments were sent for sequencing to Macrogen (Korea). M13 forward primer was used for sequencing. The sequences were trimmed from vector sequences and analysed for the presence of forward and reverse primer sites. The sequences were BLAST searched at NCBI to find their homology with sequences in the NCBI database. Later on the putative biotic stress resistance genes were also BLAST searched in the sequenced *gossypium* genomes at CottonGen (www.cottongen.org) to determine their chromosomal location.

### Retrieval of R gene sequences and primer design

Cotton RGAs of the NBS-LRR class reported by Azhar *et al.* (2010) were used to design specific primers. Gene sequences representing all five classes of R genes (including N, L6, RPP5, I2, RPS2, RPMI, Cf9, Xa21, RPW8, RRS1R, Ve1 and Pto) were retrieved from GenBank and BLAST searched in *G. arboreum* ESTs. The homologous ESTs were translated into amino acid sequences to find the open reading frame (ORF). The protein sequences of RGAs in *G. arboreum* and their homologues in *G. hirsutum* were aligned using the ClustalW program. Only those *G. arboreum* ESTs that differed from their homologues in *G. hirsutum* were selected. Primers were designed from the coding regions of ESTs using Beacon Designer software. Primer parameters were set as follows: primer length 18–24 nucleotides, GC content 40–50 %, melting temperature 55–60 °C and amplicon size 200–250 bp. The list of primers designed using the NBS class of previously reported RGAs is shown in **Supporting Information—Table S2**. Primers designed from the ESTs homologues of disease resistance genes are listed in **Supporting Information—Table S3**.

### Plant material and RNA isolation

Six cotton genotypes were used for the study, including one from *G. arboreum* (Ravi, resistant to CLCuV) and five from *G. hirsutum*, namely Coker (highly susceptible to CLCuV), NIBGE-2, NIBGE-115, N-253 and IR-3701 (tolerant to CLCuV). Young top leaf samples of CLCuV-susceptible and -non-susceptible plants (40–45 days old) of each genotype were taken from the National Institute for Biotechnology and Genetic Engineering (NIBGE) cotton field. Leaf samples were ground in liquid nitrogen and Invitrogen RNA purification reagent was used to isolate total RNA. The quality of RNA was checked by running on 1 % agarose gel. High-quality RNA was used to make first-strand cDNA using oligo dT primers. The cDNA was stored at −70 °C until further use.

### RT–PCR analysis

The concentration of the cDNA was measured on a spectrophotometer and equal concentrations were prepared for the reaction mixture. The RT–PCR reaction consisted of 5 µL of cDNA template at an appropriate dilution, 5 µL of 10× (NH_4_)_2_SO_4_*Taq* buffer, 4 µL of 25 mM MgCl_2,_ 1 µL of 10 mM dNTPs, 1 µL of each 50 ng µl^−1^ forward and reverse primer, 1 µL of 2 units of *Taq* DNA polymerase and 36 µL of PCR water. PCR was performed on a BioRad thermal cycler and the PCR profile was set as: first denaturation at 94 °C for 4 min, followed by 40 cycles of denaturation at 94 °C for 1 min, annealing at 50 °C for 1 min, extension at 72 °C for 30 s, and last cycle of final extension at 72 °C for 10 min.

## Results

### Analysis of PCR products amplified with degenerate primers

The use of degenerate primers in PCR with genomic DNA gave multiple amplifications with a single primer in most reactions. The fragments appearing in CLCuV-resistant *G. arboreum* and partly tolerant *G. hirsutum* genotypes that differed with respect to totally susceptible Coker were selected and cloned for sequencing. In this way 38 fragments were cloned from *G. arboreum* and *G. hirsutum*. The details of the cloned fragments including their appearance in the different cotton genotypes and BLASTX results are presented in Table 1.

**Table 1.** Details of the clones amplified in PCR with degenerate primers.

Clone no.	Primer name	Cloned from	Fragment length (bp)	Presence in *G. arboreum*	Presence in partially tolerant *G. hirsutum* genotypes	Presence in Coker	BLASTX results (% similarity, homology)	Role reported in literature
RM1	NLLF1 NLLR1	*G. arboreum*	432	P	No	No	89 %, TMV resistance protein N-like of *G. raimondii* (XP012434806)	Disease resistance
RM2	NLLF1 NLLR1	*G. arboreum*	430	P	P	P	84 %, ubiquitin protein ligase of *Theobroma cacao* (XM007026959)	Protein degradation in proteasomal pathway
RM3	TNLS1F3 TNLS1R2	*G. arboreum*	211	P	P	P	98 %, upstream of FLC-like transcript of *G. raimondii* (XM012636771)	Flowering response
RM4	TNLS1F3 TNLS1R2	NIBGE-115	812	P	P	No	87 %, NBRI clone microsatellite sequence of *G. hirsutum* (JX597733)	Chromosomal region having repeated sequences
RM5	TNLS1F3 TNLS1R2	NIBGE-115	468	P	P	No	72 %, *G. hirsutum* NBRI clone microsatellite sequence (JX607684)	Chromosomal region having repeated sequences
RM6	TNLS1F3 TNLS1R2	*G. arboreum*	363	P	P	No	Receptor-like protein kinase of *J. curcas* (XM012212994)	Cell surface receptor
RM7	TNLS1F3 TNLS1R1	*G. arboreum*	553	P	P	P	78 %, HR-like lesion inducing protein of *T. cacao* (XP007035055)	Unknown
RM8	TNLS1F3 TNLS1R2	*G. arboreum*	411	P	P	P	94 %, toll/interleukin receptor-1-like protein, *G. raimondii* (XP012434493)	Disease resistance
RM9	TNLS1F3 TNLS1R2	*G. arboreum*	805	P	P	No	64 %, G protein-coupled receptor of *G. arboreum* (KHG12229)	Cell surface receptors
RM10	TNLS1F1 TNLS1R1	*G. arboreum*	810	P	P	P	Unknown protein	–
RM11	TNLS1F1 TNLS1R1	*G. arboreum*	373	P	P	P	90 %, gypsy-type retroelement of *G. hirsutum* (AY395704)	–
RM12	TNLS1F3 TNLS1R2	NIBGE-115	363	P	P	No	89 %, receptor-like protein kinase of *J. curcas* (XM012212994)	Cell surface receptor
RM13	TNLS1F3 TNLS1R2	*G. arboreum*	119	P	P	No	81 %, uncharacterized protein of *T. cacao* (XM007043737)	–
RM14	TNLS1F3 TNLS1R2	*G. arboreum*	511	P	No	No	Unknown protein	–
RM15	TNLS1F3 TNLS1R2	NIBGE-115	186	P	P	No	62 %, uncharacterized protein of *G. raimondii* (XP012487892)	–
RM16	TNLS1F3 TNLS1R2	*G. arboreum*	431	p	P	P	78 %, ATP-dependent zinc metalloprotease of *G. arboreum* (KHG13773)	Mitochondrial protein metabolism
RM17	TNLS1F3 TNLS1R1	*G. arboreum*	290	P	P	P	Unknown protein	–
RM18	TNLS1F3 TNLS1R1	*G. arboreum*	547	P	P	P	48 %, protein efr3 of *G. arboreum* (KHG08222)	Plasma membrane immune receptor
RM19	TNLS1F3 TNLS1R1	*G. arboreum*	681	P	P	P	97 %, translation initiation factor IF-2 of *G. arboreum* (KHG16568)	Protein synthesis
RM20	TNLS1F3 TNLS1R1	*G. arboreum*	390	P	P	No	Unknown protein	–
RM21	TNLS1F1 TNLS1R1	NIBGE-115	176	P	P	No	60 %, uncharacterized protein of *G. raimondii* (XP012468905)	–
RM22	TNLS1F1 TNLS1R1	NIBGE-115	164	P	P	No	Unknown protein	–
RM23	TNLS1F1 TNLS1R1	NIBGE-115	223	P	P	No	100 %, hypothetical protein F383 of *G. arboreum* (KHG15854)	–
RM24	TNLS1F1 TNLS1R1	NIBGE-115	893	P	P	No	87 %, hypothetical protein B456 of *G. raimondii* (KJB57098)	–
RM25	TNLS1F1 TNLS1R1	NIBGE-115	360	P	P	No	33 %, predicted titin- like of *Maylandia zebra* (XP012771208)	Elastic protein of muscles
RM26	TNLS1F1 TNLS1R1	NIBGE-115	503	P	P	No	67 %, integrase of *G. hirsutum* (AAP43917)	Region found in retrotransposons
RM27	TNLS1F4 TNLS1R2	*G. arboreum*	461	P	P	P	89 %, hypothetical protein F383 of *G. arboreum* (KHG23397)	–
RM28	TNLS1F4 TNLS1R2	*G. arboreum*	453	P	P	P	Unknown protein	–
RM29	TNLS1F4 TNLS1R2	*G. arboreum*	808	P	P	P	48 %, uncharacterized protein of *Glycine max* (XP006599832)	–
RM30	TNLS1F6 TNLS1R6	NIBGE-115	808	P	P	No	43 %, hypothetical protein L484 of *Morusnotabilis* (XP010105619)	–
RM31	STKC3F1 STKC3R1	NIBGE-115	495	P	P	No	92 %, Ser/Thr protein kinase of *G. raimondii* (KHG29279)	Cell surface receptor
RM32	STKC5F1 STKC5R1	*G. arboreum*	344	P	P	P	76 %, ubiquitin carboxyl terminal hydrolase 22 of *T. cacao* XP007011685	Deubiquitinating enzyme
RM33	STKC5F1 STKC5R2	NIBGE-115	218	P	No	No	Unknown protein	–
RM34	STKC5F1 STKC5R2	NIBGE-115	93	P	P	P	77 %, hypothetical protein F383 of *G. arboreum* (KHG08347)	–
RM35	STKC4F STKC41	NIBGE-115	480	P	P	No	52 %, hypothetical protein L484 of *Morusnotabilis* (XP010105619)	–
RM36	LRRBF1 LRRBR1	NIBGE-115	366	P	P	No	38 %, hypothetical protein of *Glycine soja* (KHN24778)	–
RM37	LRRCF LRRCR	*G. arboreum*	606	P	P	P	100 %, methyl transferase NSUN5 of *G. arboreum* (KHG28375)	Involved in RNA metabolism
RM38	LRREF LRRER	*G. arboreum*	187	P	P	P	100 %, hypothetical protein F383 of *G. arboreum* (KHG15854)	–

The sequences of cloned fragments belonged to a variety of different pathway genes and uncharacterized proteins. However, five fragments (RM1, RM6, RM8, RM12 and RM31) showed similarity with R genes. RM1, RM6 and RM8 were cloned from *G. arboreum* and RM12 and RM13 were cloned from *G. hirsutum* genotype NIBGE-115. RM1 belongs to the NBS class and showed homology with TMV resistance like protein N. Maximum homology was found with a *G. raimondii* gene. RM6 showed homology with the receptor-like protein kinase of *Jatropha curcas*. RM8 showed the presence of TIR-2 family signatures in BLASTX results and homology with the TIR class of proteins in different plant species. Maximum homology (94 %) was observed with *G. raimondii* toll/interleukin receptor-like protein. RM12 and RM31 showed homology with the serine/threonine protein kinase class of cell surface receptors of *G. raimondii.*

### BLAST of putative R genes in sequenced *Gossypium* genomes

The identified putative RGAs were BLAST searched (at cottongen.org) in the sequenced genomes of *Gossypium* species to determine their chromosomal location (Table 2). All the RGAs showed homology with more than one chromosome, and multiple hits were observed on each chromosome for each RGA.

**Table 2.** BLAST results in the sequenced cotton genomes.

RGA name (accession no.)	*G. arboreum*	*G. hirsutum*	*G. raimondii*	R genes in *A. thaliana*
RM1 (KT250635)	Chr. no. 4, 6	Chr. no. 1, 6	Chr. no. 7, 12	150
RM6 (KT886994)	Chr. no. 11	Chr. no. 3, 5	Chr. no. 13	610
RM8 (KT633945)	Chr. no. 4, 6	Chr. no. 1, 6	Chr. no. 7, 12	150
RM12 (KT885194)	Chr. no. 5, 9, 11	Chr. no. 6, 11, 13	Scaffold 430, 181	610
RM31 (KT633946)	Chr. no. 5, 9, 8, 11	Chr. no. 3, 9, 11	Chr. no. 5, 6, 7, 9, 11	610

### RT–PCR for evaluating RGA expression

#### Expression analysis of NBS class of R gene homologues.

**Figure 1.1–1.7.**
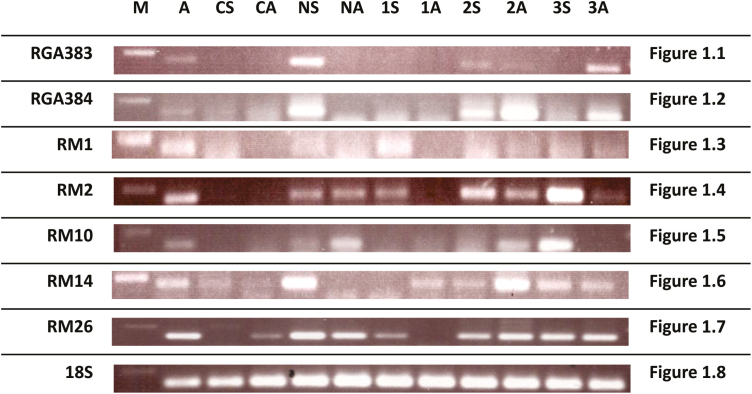
RT–PCR results are shown here for RGA 383, RGA 384, RM01, RM02, RM10, RM14 and RM26. These RGAs and ESTs are showing variation in expression among the CLCuV-resistant *G. arboreum* and tolerant *G. hirsutum* genotypes and little or no expression in susceptible Coker. Figure 1.8: Similar quantities of cDNA (ng/microliter) were tested with 18S housekeeping gene primers as internal control. The abbreviations mentioned in figures are explained as follows: A = *G. arboretum*; CS = symptomatic Coker; CA = asymptomatic Coker; NS = NIBGE-2 symptomatic; NA = NIBGE-2 asymptomatic; 1S = NIBGE-115 symptomatic; 1A = NIBGE-115 asymptomatic; 2S = 253 symptomatic; 2A = 253 asymptomatic; 3S = IR-3701 symptomatic; 3A = IR-3701 asymptomatic; − = negative PCR; M = 1 Kb DNA marker.

The expression of 24 RGAs of the NBS class reported by Azhar *et al.* (2010) was studied in cotton genotypes. RT–PCR analysis showed that RGA 379, 383, 384, 395 and 401 were expressed in *G. arboreum* as well as *G. hirsutum* genotypes (Table 3). The expression of RGAs was variable in all tested genotypes and differed between symptomatic and asymptomatic plants, with the exception of RGA 395, whose expression level was the same in both diseased and healthy plants. Resistance gene analogue 375, 378, 381, 385, 390, 394, 411, 414, 418 and 421 showed no expression in *G. arboreum* or any of the genotypes of *G. hirsutum*. Five RGAs showed high to moderate expression in Ravi. There were similar expression patterns in both symptomatic and asymptomatic plants of Coker. In NIBGE-2 most of the RGAs were expressed in symptomatic plants but not in asymptomatic plants. In the case of NIBGE-115 only one RGA (RGA 395) was expressed in both symptomatic and asymptomatic plants. Relatively more RGAs were expressed in asymptomatic plants of N-253 and IR-3701 compared with symptomatic plants. Resistance gene analogue 383 and 384 seem important as they showed almost no expression in either symptomatic or asymptomatic coker plants (CLCuV susceptible), as seen in Fig. 1.1 and 1.2.

**Table 3.** Expression analysis of NBS-LRR class of RGAs in *G. arboreum* and *G. hirsutum* genotypes under CLCuV infestation.

S. no.	RGAs	RGA accession no.	*G. arboreum* (Ravi)	*G. hirsutum* (Coker)		*G. hirsutum* (NIBGE-2)		*G. hirsutum* (NIBGE-115)		*G. hirsutum* (N-253)		*G. hirsutum* (IR-3701)	
				Symptomatic	Asymptomatic	Symptomatic	Asymptomatic	Symptomatic	Asymptomatic	Symptomatic	Asymptomatic	Symptomatic	Asymptomatic
1	372	FM992081	–	–	–	++	–	–	–	++	–	–	–
1	373	FM992082	–	–	–	+++	–	–	–	+++	–	–	+++
3	375	FM992083	–	–	–	–	–	–	–	–	–	–	–
4	377	FM992084	–	+	++	+++	–	–	–	–	++	–	–
5	378	FM992085	–	–	–	–	–	–	–	–	–	–	–
6	379	FM992086	+++	+++	+++	+++	+++	–	–	–	+++	+++	+++
7	381	FM992087	–	–	–	–	–	–	–	–	–	–	–
8	383	FM992088	++	–	–	++	–	–	–	+	+	–	++
9	384	FM992089	++	+	–	+++	–	–	–	+++	+++	–	+++
10	385	FM992090	–	–	–	–	–	–	–	–	–	–	–
11	390	FM992091	–	–	–	–	–	–	–	–	–	–	–
12	394	FM992092	–	–	–	–	–	–	–	–	–	–	–
13	395	FM992093	+++	+++	+++	+++	+++	+++	+++	+++	+++	+++	+++
14	401	FM992094	+++	++	++	+++	–	–	–	+++	+++	+++	+++
15	411	FM992095	–	–	–	–	–	–	–	–	–	–	–
16	412	FM992096	–	–	–	+++	–	–	–	–	+	–	–
17	413	FM992097	–	–	–	–	–	–	–	–	–	–	–
18	414	FM992098	–	–	–	–	–	–	–	–	–	–	–
19	415	FM992099	–	++	+	+++	++	–	–	+++	+	+	+
20	416	FM992100	–	+	+	+++	–	–	–	+++	+	+++	+
21	417	FM992101	–	–	–	–	–	–	–	–	–	–	–
22	418	FM992102	–	–	–	–	–	–	–	–	–	–	–
23	420	FM992103	–	–	–	+	–	–	–	–	+++	+++	++
24	421	FM992104	–	–	–	–	–	–	–	–	–	–	–
18S			+++	+++	+++	+++	+++	+++	+++	+++	+++	+++	+++

+++ = high expression; ++ = moderate expression; + = low expression; – = no expression.

#### RT–PCR for evaluating expression of EST homologues of R genes.

The *G. arboreum* ESTs were searched in GenBank against the various R gene classes. No ESTs showed similarity to the NBS class of R genes. However, EST homologues were found against the serine/threonine kinase (STK) and LRR class of genes. One EST was also found showing similarity with Rar1 (R gene-dependent signalling molecule). The selected ESTs have conserved domains of PKc-STKc, LRR-8 and CHORD as found in the BLASTp search (Table 4). The ESTs were further used for BLASTx search to determine their similarity with other genes in different species. The *E*-value and percent similarity were significant, inferring that the selected ESTs were representative of the R genes.

**Table 4.** Details of ESTs retrieved from GenBank along with their BLASTP and BLASTX results.

*G. arboreum* ESTs		BLATP results		BLASTX results				
S no.	Accession no.	Conserved domain	Protein length	Species	Accession no.	Gene description	*E*-value	% Similarity
1	BG445606	PKc-STKc	108	*Cucumissativus*	XP_004147149	Predicted STK	2e-51	80
2	BE052979	PKc-STKc	235	*Camellia sinensis*	ABG81507	STK	2e-94	83
3	BG445231	PKc-STKc	254	*Gossypiumhirsutum*	AGH08169	CBL-interacting kinase protein	3e-131	89
4	DT552125	PKc-STKc	282	*Gossypiumhirsutum*	AAT64036	Putative STK	0.0	100
5	BE053080	PKc-STKc	206	*Ricinuscommunis*	XP-002517716	STK	6e-75	68
6	BG440641	PKc-STKc	163	*Theobroma cacao*	EOY04490	CBL-interacting kinase protein	1e-84	72
7	JG857090	Pkc	142	*Arachishypogaea*	AFB69787	Pto resistance protein	6e-119	91
8	FG548152	PKc-SPS1	148	*Theobroma cacao*	EOY23503	Kinase superfamily protein	1e-74	83
9	BM360121	PKc	168	*Dasypyrumvillosum*	AEF30546	STK	6e-108	97
10	BQ414633	PKc-STKc	183	*Medicago sativa*	AAN63591	GSK-3-like protein	5e-127	97
11	BF273477	PKc-SPS1	111	*Theobroma cacao*	EOX96762	STK	2e-52	72
12	BQ412362	LRR-8	108	*Populous trichocarpa*	XP002311609	LRR protein	6e-34	75
13	BG447461	LRR-8	135	*Theobroma cacao*	EOY34306	LRR protein	6e-88	86
14	BG441980	PKc-RLK	210	*Citrus trifoliate*	ABY40731	FERONIA receptor-like kinase	4e-120	93
15	BF269236	LRR-8	201	*Populous trichocarpa*	XP-002301687	LRR transmembrane protein	3e-62	62
16	BF273652	LRR-8	175	*Populous trichocarpa*	XP-002301687	LRR transmembrane protein	2e-67	66
17	BQ407203	PKc	210	*Theobroma cacao*	EOY04413	LRR-kinase family protein	6e-119	87
18	BF271799	PKc	177	*Ricinuscommunis*	XP002511696	Putative LRR receptor kinase	3e-66	74
19	BF273688	PKc	63	*Ricinuscommunis*	XP002527897	Putative kinase	1e-65	66
20	BQ411434	PKc	220	*Ricinuscommunis*	XP-002510817	LRR receptor protein kinase	2e-115	82
21	BG445718	PKc	296	*Populous trichocarpa*	XP-002301687	LRR transmembrane protein	2e-138	79
22	BQ405546	PKC-LRR-STK	212	*Populustremula*	AGS56396	LRR receptor-like STK	2e-134	92
23	BG441233	LRR-8	204	*Gossypiumhirsutum*	ACD93187	Polygalacturonase-inhibiting protein	1e-129	88
24	JG854980	LRR-8	280	*Gossypiumbarbadense*	AAQ19807	Polygalacturonase-inhibiting protein	2e-167	92
25	BQ403907	LRR-RLPK	191	*Gossypiumbarbadense*	AAQ19807	Polygalacturonase-inhibiting protein	7e-83	98
26	BF276058	CHORD	201	*Ricinuscommunis*	XP002510896	Putative rar1	3e-111	72
27	BQ407587	PKc	166	*Ricinuscommunis*	XP-002520123	STK, PBS1	2e-73	74
28	BF269489	PKc	182	*Arabidopsis thaliana*	NP178291	Putative receptor-like kinase	2e-142	80

The primer design was based on *G. arboreum* ESTs and thus it was expected that all primers would result in amplification in at least *G. arboreum*. However, the RT–PCR results (Table 5) indicated that primers for RM3, RM20, RM22 and RM27 did not result in amplification in *G. arboreum* or in genotypes of *G. hirsutum*. All other ESTs were expressed in *G. arboreum* and *G. hirsutum* genotypes. RM1, RM2, RM10, RM14 and RM26 (homologues of STKs, RLKs and Rar1 resistance genes) are important ESTs as they are expressed in *G. arboreum* and tolerant *G. hirsutum* genotypes, whereas no expression was detected in susceptible Coker (Fig. 1.3–1.7). The ESTs expressed in *G. arboreum* were also expressed in all genotypes of *G. hirsutum* except for RM28, which was expressed at low levels only in some *G. hirsutum* genotypes. The expression level of ESTs ranged from low to high in Ravi. Relatively high EST expression was observed in symptomatic and asymptomatic plants of Coker. The expression pattern of ESTs was similar and high in symptomatic and asymptomatic plants of NIBGE-2, N-253 and IR-3701. Higher expression levels were detected in NIBGE-115, with more ESTs in symptomatic plants compared with asymptomatic ones.

**Table 5.** RT–PCR based expression analysis of ESTs in *G. arboreum* and *G. hirsutum* genotypes under CLCuV infestation.

ESTs names	*G. arboreum* ESTs accession no.	*G. arboreum*	*G. hirsutum* (Coker)		*G. hirsutum* (NIBGE-2)		*G. hirsutum* (NIBGE-115)		*G. hirsutum* (N-253)		*G. hirsutum* (IR-3701)	
			Symptomatic	Asymptomatic	Symptomatic	Asymptomatic	Symptomatic	Asymptomatic	Symptomatic	Asymptomatic	Symptomatic	Asymptomatic
RM01	BG445606	++	–	–	+	–	++	–	–	–	–	+
RM02	BE052979	++	–	–	+	+	+	–	++	+	+++	+
RM03	BG445231	–	–	–	–	–	–	–	–	–	–	–
RM04	DT552125	+	++	++	++	++	+++	+	+++	+++	+++	+++
RM05	BE053080	+++	–	++	+++	+++	+++	–	+++	+++	+++	+++
RM06	BG440641	+++	+	+++	+++	+++	+++	–	+++	+++	+++	+++
RM07	JG857090	+++	+	+++	+++	+++	+++	–	+++	+++	+++	+++
RM08	FG548152	++	+	+	+++	+++	++	++	+++	+++	+++	+++
RM09	BM360121	++	++	–	+++	+++	+++	++	+++	++	+++	+++
RM10	BQ414633	++	–	–	+	++	–	–	–	++	+++	–
RM11	BF273477	+++	+	+	+++	+++	+++	++	+++	+++	+++	++
RM12	BQ412362	+++	+	++	+++	+++	+++	–	+++	+++	+++	+++
RM13	BG447461	+++	+	+	+	+	++	+	++	+++	++	+++
RM14	BG441980	++	+	–	+++	–	–	+	+	+++	+	+
RM15	BF269236	+++	++	++	+++	+++	++	++	+++	+++	+++	++
RM16	BF273652	+++	+	+++	+++	+++	++	–	+++	+++	+++	+++
RM17	BQ407203	+++	+	+	+++	+++	+++	–	++	+++	+++	+++
RM18	BF271799	+++	++	+++	+++	+++	+++	+	+++	+++	+++	+++
RM19	BF273688	+++	+	+	+++	++	+++	++	+++	+++	+++	+++
RM20	BQ411434	–	–	–	–	–	–	–	–	–	–	–
RM21	BG445718	+++	+	+	+++	+++	+++	+	++	++	+++	++
RM22	BQ405546	–	–	–	–	–	–	–	–	–	–	–
RM23	BG441233	+	+	–	++	++	++	–	+	+++	+++	++
RM24	JG854980	+	+	–	++	++	+++	–	++	+++	–	+
RM25	BQ403907	+++	+	+++	+++	+++	+++	–	+++	+++	+++	+++
RM26	BF276058	+++	–	+	+++	+++	+	–	+++	+++	+++	+++
RM27	BQ407587	–	–	–	–	–	–	–	–	–	–	–
RM28	BF269489	–	–	–	–	–	+	–	–	+	+	+
18S		+++	+++	+++	+++	+++	+++	+++	+++	+++	+++	+++

+++ = high expression; ++ = moderate expression; + = low expression; – = no expression.

## Discussion

Degenerate PCR primers can be used to isolate genes from species on the basis of conserved domains within the genes. This approach has been used in cotton. Gao *et al.* (2006) cloned RGAs of the NBS class and also defence gene analogues from *G. barbadense* using the degenerate primer approach. Similarly, Tan *et al.* (2003) reported the cloning of 33 RGAs from *G. hirsutum* using degenerate primers based on conserved domains of NBS motifs. In this study, degenerate primers were designed using the conserved regions of nucleotide sequence alignments of the reported plant R genes of various classes deposited in GenBank. The PCR amplified 38 fragments from genomic DNA using degenerate primers, and these were cloned from *G. arboreum*, *G. hirsutum* and the genotype NIBGE-115. Out of these amplified fragments, five fragments corresponded to RGAs encoding the TIR and STK classes of R genes. Resistance gene analogues from cotton belonging to the NBS class have also been reported (Tan *et al.* 2003; Gao *et al.* 2006).

Prior sequence information and the annealing of primers at multiple complementary but non-specific sites are some of the limitations of PCR (Garibyan and Avashia 2013). In this study, PCR on genomic DNA using degenerate primers resulted in non-specific amplifications due to non-specific primer binding. The forward and reverse primer sequences were observed in almost all of the cloned fragments; however, few of them represented R genes.

*Gossypium arboreum* has a very sturdy defence mechanism to combat CLCuV (Khan *et al.* 2016). The ESTs identified from *G. arboreum* show conserved domains of PKc-STKc (ESTs No. 1, 2, 3, 4, 5, 6, 10, 14, 22 contained PKc-STKc domains and ESTs 7, 9, 17, 18, 19, 20, 21, 27, 28 contained only the PKc domain) while the LRR domain was represented by seven ESTs (ESTs No. 12, 13, 15, 16, 23, 24 and 25). PKc is a member of the AGC group in the protein kinase superfamily (Newton and Messing 2010). These enzymes are involved in the phosphorylation of serine and threonine amino acids found in proteins (Trotta *et al.* 2016). Serine/threonine kinase is one of the PKc enzymes involved in the regulation of cell proliferation, programmed cell death (apoptosis), cell differentiation and embryonic development (Newton and Messing 2010; Trotta *et al.* 2016). The BLASTX results indicated that the *G. arboreum* ESTs contained PKc-like domains and had similarity with STKs and the LRR-kinase class of genes in other crop plants. Two *G. arboreum* ESTs had domains of PKc-SPS1 (ESTs No. 8, 11). This is an important class of kinases that are known to activate the p38 MAP kinase pathway of stress-induced signal transduction in mammals. The presence of a PKc-like domain in *G. arboreum* ESTs was interesting and it can be inferred that these ESTs were from the protein kinase class of genes in *G. arboreum*.

Leucine-rich repeat receptor kinases (LRR-RKs) are the largest subfamily of transmembrane receptors, with over 200 members in *Arabidopsis* (Shiu and Bleecker 2001). Leucine-rich repeat receptor kinases are mediators of plant defence and developmental processes. Some ESTs in this study (ESTs No. 12, 13, 15, 16, 23 and 24) contained the domain for LRR-8, which is an important domain of LRR-RK proteins that plays a critical role in ligand recognition in the CLV1 and BAM family of cell receptors (Shinohara *et al.* 2012). Expressed sequence tags 23, 24 and 25 containing LRR domains showed significant homology with polygalacturonase-inhibiting protein (PGIP) in the BLASTX results. Plant PGIPs help confer resistance against cell wall-degrading phytopathogens like fungi, bacteria and nematodes (Juge 2006; Kikot *et al.* 2009; Danchin 2011).

One EST (26) showed significant similarity with the resistance signalling gene *Rar*1 bearing the zinc binding CHORD domain that is found in all eukaryotes except yeast. Muskett *et al.* (2002) reported that Rar1 acts as a rate-limiting factor for R gene triggered defence activation. It regulates the extent of pathogen containment, hypersensitive plant cell death and oxidative burst at primary infection sites. So the selected ESTs were representative of the important genes in plant defence mechanisms.

Resistance gene analogues have been used to study the genetics of resistance in plant species using R gene specific or degenerate primers (Leister *et al.* 1996; Chen *et al.* 1998). Azhar *et al.* (2010) identified 24 RGAs of the NBS class in cotton and utilized these RGAs as markers in interspecific hybrid selection for analysing *G. arboreum* genome contribution. In the present study, RGAs were used to reveal the expression pattern in diploid and tetraploid cotton. Expression analysis of cell surface receptors was also conducted by utilizing their conserved domain regions of LRR and STK collected from *G. arboreum* ESTs. The selected ESTs were confirmed to contain the LRR and STK domains of resistance genes through BLASTX and DELTA-BLAST search as shown in Table 4. ESTs represent the more conserved part of the genome and EST-based markers can be studied in other species of cotton (Scott *et al.* 2000). *Gossypium arboreum* ESTs were not found in the BLAST search of the NBS class of genes. The expression of STK and LRR ESTs was higher than that of the NBS class of RGAs. It can be deduced that in *G. arboreum* R genes of classes other than NBS-LRR might be more active to counter biotic and abiotic stresses. Besides the importance of the NBS class of R genes, transmembrane receptors might also play a role in the recognition of and defence against CLCuV in *G. arboreum*. The kinase domain of disease resistance genes might play a role in phosphorylation and thus help transfer phosphate from the high-energy phosphate donor molecules.

Using the same set of primers designed using *G. arboreum* ESTs, amplification in RT–PCR was seen in both *G. arboreum* and *G. hirsutum*. However, there was a variable level of expression among the tested genotypes. Expression of functional genes is mostly variable in the sense that some genes are constitutively expressed and some are induced only in particular conditions and thus expression may be stage specific. Resistance gene analogues that are not expressed at all might be non-functional as they were identified from genomic DNA. Expressed sequence tags with no expression in the genotypes under study might be influenced by the environment, and these may also be stage or tissue specific, because cotton ESTs from the NCBI were mainly fibre specific. The RGAs and ESTs expressed in *G. hirsutum* but not *G. arboreum* might be D-genome specific. The RGAs and ESTs expressed in both *G. arboreum* and *G. hirsutum* may be A-genome specific.

Resistance gene analogues and ESTs expressed only in *G. arboreum* and in asymptomatic plants of *G. hirsutum* could be useful in the study of resistance against CLCuV. Resistance gene analogues and ESTs not expressed in Coker might be helpful in the study of CLCuV resistance. In this context, RGA 383 and 384 of the NBS-LRR class and the ESTs RM1, RM2, RM10, RM14 and RM26 (homologues of STKs, RLKs and Rar1 resistance genes) may be important for further studies on CLCuV resistance and the role of the kinase domain of R genes under similar conditions of inoculum and biotic stresses. As mentioned earlier these genes are known to be important players in plant defence, especially in the context of causing programmed cell death in infected tissues. So these RGAs and ESTs may be useful for isolating full-length genes, and their particular functions should be explored in response to biotic or abiotic stresses.

## Supporting Information

The following additional information is available in the online version of this article—

**Table S1.**

**Table S2.** The list of primers designed on nucleotide-binding site (NBS) class of previously reported resistance gene analogues (RGAs).

**Table S3.** The list of primers designed from the expressed sequence tag (EST) homologues of disease resistance genes.

Supplemetary Data

## Acknowledgements

This research work was funded through Indigenous 5000 PhD fellowship program of Higher Education Commission, Government of Pakistan awarded to Rakhshanda Mushtaq at National Institute for Biotechnology and Genetic Engineering (NIBGE), Faisalabad in affiliation with Pakistan Institute of Engineering and Applied Sciences (PIEAS) Nilore Islamabad, Pakistan.

## Contributions by the authors

R.M. solely done the experiments and K.S. helped in gene transformation experiments. Z.H.S., T.M. and Z.A. helped in bioinformatics tools and analysis and research write up. H.A., Y.Al-Z. and H.A.S.A. provided lab space for some of the experiments and helped in expression analysis.

A.B. was the Principal Investigator of the research project. S.M. is the group leader and helped in project planning and execution of the experiments.
