# Supplementary material for: Exploration of cotton leaf curl virus resistance genes and their screening in Gossypium arboreum by targeting resistance gene analogues
Source: AoB Plants. 2018 Oct 16;10(6):ply067. doi: 10.1093/aobpla/ply067 (PMC6247833; doi:10.1093/aobpla/ply067)
Supplement: Supplemetary Data [file ply067_suppl_supplemetary_data.docx]

Supplemetary Data:

**Supplementary Table 1**

| S.No | Primer | Sequence | Length |
| --- | --- | --- | --- |
| 1 | TNLS1F1 | 5' AGATTGYYRCAGATGTTTCA 3' | 20 mer |
| 2 | TNLS1F2 | 5' ACCDTCRAGGGATTTTGA 3' | 18 mer |
| 3 | TNLS1F3 | 5' AGATGATTGGMATTTGGGGTC 3' | 21 mer |
| 4 | TNLS1F4 | 5' GTAAGWCTACCATTGCTAGA3' | 20 mer |
| 5 | TNLS1R1 | 5' AGACAKARGATCTCAAGAGC3' | 20 mer |
| 6 | TNLS1R2 | 5' TKGCAASCTCTTCAAAACCATC 3' | 22 mer |
| 7 | TNLS1F5 | 5' TTYCTTCAYATTGCATGCTT 3' | 20 mer |
| 8 | TNLS1R5 | 5' TYNKAGGAAGACTTTTTCT 3' | 19 mer |
| 9 | TNLS1F6 | 5' RMWGCMGAAGCCTCAAGA 3' | 18 mer |
| 10 | TNLS1R6 | 5' WMAWGATCTKTACACCACA3' | 19 mer |
| 11 | TNLS2F1 | 5' GAGAAGCGAAAATGGATGCG 3' | 20 mer |
| 12 | TNLS2R1 | 5' TGCTGTATAAAGCTCTTCCT 3' | 20 mer |
| 13 | TNLS2F2 | 5' GTTCAAATTCCGAGCTTCA 3' | 19 mer |
| 14 | TNLS2R2 | 5' TTGAGCAGCACTCACTATAG3' | 20 mer |
| 15 | TNLS3F 5' | CTTYATATAGCWTGYYTTTTCAAC 3' | 24 mer |
| 16 | TNLS3R | 5' TRGCWSTTGAGAGATCAGG 3' | 19 mer |
| 17 | TNLS4F | 5' TGGTGCTTRRATGAATTGGT 3' | 20 mer |
| 18 | TNLS4R1 | 5' SCKATRGTASTCTTACCAAT 3' | 20 mer |
| 19 | TNLS4R2 | 5' GRTCACRAYWATTCTGCTTCCA 3' | 22 mer |
| 20 | TNLS5F' | 5'GAYGTVTTCDYRAGCTTCAG 3' | 20 mer |
| 21 | TNLS5R | 5' WKATCATCCAKATCATCAAT3' | 20 mer |
| 22 | NTNLF1 | 5' GAAGACGACACTTGCTCAG 3' | 19 mer |
| 23 | NTNLR1 | 5'GCTCCCAATCATCAGGTTTAG 3' | 21 mer |
| 24 | NTNLF2 | 5' CAGTTTCGTGTCGGGTTG 3' | 18 mer |
| 25 | NTNLR2 | 5' ACAATGATAATAAGGTGGAGGAG 3' | 23 mer |
| 26 | CNLS1F | 5' CCAGAGATATGCCAGTTG 3' | 18 mer |
| 27 | CNLS1R | 5' TGTTGTTGTTATCATCAGAC 3' | 20 mer |
| 28 | CNLS2F | 5' TTTGCATGACATGATGAGAG 3' | 20 mer |
| 29 | CNLS2R | 5' AAGTATCKYAAGTGGATGAG 3' | 20 mer |
| 30 | CNLS3F1 | 5' ATGTTGGARCRAACTCGACA 3' | 20 mer |
| 31 | CNLS3R1 | 5' GACAMSAKAAGTRTCTCTAAG 3' | 21 mer |
| 32 | CNLS3R2 | 5' CWABAATTGATAGAGATCC 3' | 19 mer |
| 33 | CNLS3F2 | 5' GCTGTTTACAAYGATGAGAA 3' | 20 mer |
| 34 | CNLS3R2 | 5' ATCARYGCTGGTAAGATTCC 3' | 20 mer |
| 35 | CNLS4F | 5' GCTCTGGGAGGTCTTCAACA 3' | 20 mer |
| 36 | CNLS4R | 5' GAGTCYGATAATTCCAGCA 3' | 19 mer |
| 37 | CNLS5F1 | 5' TWCCKATTCTMAAGTACAGC3' | 20 mer |
| 38 | CNLS5R1 | 5' CWCGRACCACRTCATGCATC 3' | 20 mer |
| 39 | CNLS5F2 | 5' YGTWTTGAASGAGYTGCAGC 3' | 20 mer |
| 40 | CNLS5R2 | 5' TKARTGGAAGYTTTCTCAGC 3' | 20 mer |
| 41 | CNLS6F | 5' GTCCCACCTTTTCCCTTCTT 3' | 20 mer |
| 42 | CNLS6R | 5' GATTCGGGGCCAGTAGTGGG 3' | 20 mer |
| 43 | CNLS7F1 | 5' HSAADTCMAGGTATGGCT 3' | 18 mer |
| 44 | CNLS7R1 | 5' BAGAYACSACAACCCAAAT 3' | 19 mer |
| 45 | CNLS7F2 | 5' GATAGAKYAYTGGATATGCGAGG 3' | 23 mer |
| 46 | CNLS7R2 | 5' ATAGATCYAAAACARCTAGC 3' | 20 mer |
| 47 | CNLS8F | 5' AGAAGARCTAGTGAGGAGAA 3' | 20 mer |
| 48 | CNLS8R | 5' AGWAGAAGGTAGATGAGATA 3' | 20 mer |
| 49 | NLLF1 | 5' GGGTGGAAAGTAGAAAGAAG 3' | 20 mer |
| 50 | NLLR1 | 5' TATGACGTCGACATAGAAAC 3' | 20 mer |
| 51 | NLLF2 | 5' GTCGATAACTTTCTTCTTTG 3' | 20 mer |
| 52 | NLLR2 | 5' CACAATTGGAAATGTCAATC 3' | 20 mer |
| 53 | NLSS1F | 5'GADHTCTCTCACCRATTTGAAG 3' | 22 mer |
| 54 | NLSS1R1 | 5' CWACATCRTCAAGRACAACTAA 3' | 22 mer |
| 55 | NLSS1R2 | 5' AAGAGCTYSAYMGCCTC 3' | 17 mer |
| 56 | NLSS3F | 5'GGGGGGGTGGGGAAGACG 3' | 18 mer |
| 57 | NLSS3R | 5' AGGGCGAGGGGGAGGCC 3' | 17 mer |
| 58 | NLSS6F | 5'CYGGATCDCATDVTTGGTTTGGT 3' | 23 mer |
| 59 | NLSS6R | 5' GAGDGGRARDCCRNYRGCATAAGA 3' | 24 mer |
| 60 | STKC1F | 5' TGCWGYTGCWGATGTTTGGTC 3' | 21 mer |
| 61 | STKC1R1 | 5' AMACAGCMTKWACATCATCCA 3' | 21 mer |
| 62 | STKC1R2 | 5' CCYTCWAYCCTCATCTTGTA 3' | 20 mer |
| 63 | STKC3F1 | 5' GGGACAAGTTATACCAAATG3' | 20 mer |
| 64 | STKC3R1 | 5' AGGCAATTCTAATGTCAGAG3' | 20 mer |
| 65 | STKC3F2 | 5' TTGCTTCATTCACCAGAG 3' | 18 mer |
| 66 | STKC3R2 | 5' TTCTCCTCGTATAGTTCC 3' | 18 mer |
| 67 | STKC4F | 5' STGAAGRTYTTGAARGA 3' | 17 mer |
| 68 | STKC4R | 5' CATYCCRTARCTRTACACATC 3' | 21 mer |
| 69 | STKC5F1 | 5' GGAGAAGAYTTCATMAATGAAAGT 3' | 24 mer |
| 70 | STKC5R1 | 5' TCCARSAGAATGTTKTGAGG 3' | 20 mer |
| 71 | STKC5R2 | 5' AYYTCTGGTGCRATGTACCC 3' | 20 mer |
| 72 | LRRAF | 5'TTGGKGARGGWGGATTTGG 3' | 19 mer |
| 73 | LRRAR | 5'AWGCCCAWTCVAGAAGGTA 3' | 19 mer |
| 74 | LRRBF1 | 5' TCYGGTGAKATTCCKGATCT 3' | 20 mer |
| 75 | LRRBR1 | 5' CCWARAAMYTCWGCAGAAGCT 3' | 21 mer |
| 76 | LRRBF2 | 5' TRYTCYAARGAHGARAAGCT 3' | 20 mer |
| 77 | LRRBR2 | 5' TCKGTTAYYTCTGGYGCACG 3' | 20 mer |
| 78 | LRRCF | 5' TCTYGACWTSCGCAACAA 3' | 18 mer |
| 79 | LRRCR | 5' GCTACMGSAGAACCATCTCT 3' | 20 mer |
| 80 | LRRDF | 5' CAAGCYACCCAATTGACTTA 3' | 20 mer |
| 81 | LRRDR | 5' TCCAASTTGTTCTTCCGGAG 3' | 20 mer |
| 82 | LRREF | 5' AWGTTTCCRTTCTGGTTGAG 3' | 20 mer |
| 83 | LRRER | 5' CGWAKCCTATTGCAGCTGC 3' | 19 mer |
| 84 | LRRFF | 5' CTCCGTTACTTGTATCTTCA 3' | 20 mer |
| 85 | LRRFF | 5' TCTTTCTACAGAGACAGAAC 3' | 20 mer |
| 86 | LRRGF1 | 5' TTYMAGTRTCRAGCTTTTGT 3' | 20 mer |
| 87 | LRRGR1 | 5' TTAGYCAATAAGCTAGCTA 3' | 19 mer |
| 88 | LRRGF2 | 5' GCTTATTGRCTAATAAAGCTA 3' | 21 mer |
| 89 | LRRGR2 | 5' CATTSAGTCKCAGGTACTTCAA 3' | 22 mer |

**Supplementary Table 2:**

**The list of primers designed on NBS class of previously reported RGAs is as follow**

|  | **Primer Names** | **Primer Sequences** | **EMBL Accession No.** |
| --- | --- | --- | --- |
| 1 | 372RTF  372RTR | TGTTTCAAATCCACCCTCTCC TAGCGTCTAAAGATTCTGATGTTG | FM992081 |
| 2 | 373RTF  373RTR | ATGTTCGTCTCTTGTCGTTAC  CCAGATATTGCTTGATGAATGC | FM992082 |
| 3 | 375RTF  375RTR | CTTCTTTCCCTAATCTTGCTTGAC  CTACTCCCTAAACCGAACCAATC | FM992083 |
| 4 | 377RTF  377RTR | TCTTGCCAGATGAATGCC  GTTGTTACAATGATCCTACTCC | FM992084 |
| 5 | 378RTF  378RTR | TCCAATTCATCAACATCATCAAG  GAGAAACAAGTCAAGATTACAATG | FM992085 |
| 6 | 379RTF  379RTR | AATGCTCATCTCTTGTTGTTAC  CTAATCTTGCTTGACGAATGC | FM992086 |
| 7 | 381RTF  381RTR | CCACCTTCTCCCATAAATC  TATTACGATGTTGGAAAGATTC | FM992087 |
| 8 | 383RTF  383RTR | AATGCTCATCTCTTGTTGTTAC  CTAATCTTGCTTGACGAATGC | FM992088 |
| 9 | 384RTF  384RTR | ATCAGGATTCAATGTTGTAGG  TCTTGTTATTCTTGATGATGTTG | FM992089 |
| 10 | 385RTF  385RTR | CACCTTCTCCCATAAATC  ATATTACGATGTTGGAAAG | FM992090 |
| 11 | 390RTF  390RTR | ATTGGTTCGGTTTAGGGAGTAG  TCTGTTGGTATTGTATCATCATCG | FM992091 |
| 12 | 394RTF  394RTR | AGAAGTTTCAGACAAATGC  TATCAAGAACAACAAGAACC | FM992092 |
| 13 | 395RTF  395RTR | AAGGTTCTTGTTGTTCTTGATG  CATTTGGATTCAGTGTTGTAGG | FM992093 |
| 14 | 401RTF  401RTR | GCATATACACATCATCAACTTG  AATTAGTCGTACATTGTCTCG | FM992094 |
| 15 | 411RTF  411RTR | ACTAACTACTCGCTCAAAGAATG  GCACACTTGGTAGCAATCTG | FM992095 |
| 16 | 412RTF  412RTR | CCTAATCTTGCTTGACGAATG  AATGCTCATCTCTTGTTGTTAC | FM992096 |
| 17 | 413RTF  413RTR | CTACAACGATCCCAGAGC  GATATTTCTTTCCCGACAGC | FM992097 |
| 18 | 414RTF  414RTR | CCTAATCTTGCTTGACGAATG  AATGCTCATCTCTTGTTGTTAC | FM992098 |
| 19 | 415RTF  415RTR | GGGCGTTGGTGTCTAAAG  CCCTCTCCCATAAATCATCC | FM992099 |
| 20 | 416RTF  416RTR | GGCTGTTGTGACTCATAC  GAATCCCTACTTCCATCAG | FM992100 |
| 21 | 417RTF  417RTR | CCTAATCTTGCTTGACGAATG  AATGCTCATCTCTTGTTGTTAC | FM992101 |
| 22 | 418RTF  418RTR | ACTAACTACTCGCTCAAAGAATG  GCACACTTGGTAGCAATCTG | FM992102 |
| 23 | 420RTF  420RTR | GTTGACCAGAAGGCTACAG  TTCCATTTCGCCACATACC | FM992103 |
| 24 | 421RTF  421RTR | ACACTCAAATGTCATCTC  TTGTTAGATAACCTGTGG | FM992104 |

**Supplementary Table 3:**

Primers designed from the ESTs homologs of disease resistance genes are listed as follows:

|  | **Primer Names** | **Primer Sequences** | **Respective *G. arboreum* ESTs**  **(Accession No.)** |
| --- | --- | --- | --- |
| 1 | RM1FP  RM1RP | GGTCACTTGGGGCAATTATG  GTTCCAAGCCTCTGCTCAAC | BG445606 |
| 2 | RM2FP  RM2RP | ATTCAGCGAGGATGAGGCTA  ATATGCGGGAGTTCCAACAG | BE052979 |
| 3 | RM3FP  RM3RP | TCCGTCATGAAAATGGTGAA  CGAGCTGCTGGAAGTAAACC | BG445231 |
| 4 | RM4FP  RM4RP | GATTCCACCTCCAAGTCCAA  AGAGATTTCGCGCTTGATGT | DT552125 |
| 5 | RM5FP  RM5RP | ACCTGTCAGGATGTTTGACG  TTATCCCACCAGAGAAAGCC | BE053080 |
| 6 | RM6FP  RM6RP | TGGATACCTACCGTCAACGA  AGAGAAGCGGTCTCTCGTGT | BG440641 |
| 7 | RM7FP  RM7RP | CCTCCATACAGGCTATGCAA  TACATCCGATTTCTCCGTCA | JG857090 |
| 8 | RM8FP  RM8RP | CTGTTGCTTGAAATGGTTGG  CATCGAAGGACGATCAACAG | FG548152 |
| 9 | RM9FP  RM9RP | TTGTGTGGAAGGAAACCAGA  TGCTTTGATATCCCGATGAA | BM360121 |
| 10 | RM10FP  RM10RP | GCAGAAGAAACCTGGGAAAG  TTGCATTCACCCATTCTTTG | BQ414633 |
| 11 | RM11FP  RM11RP | TCATGTTCGGAATGTTATTGC  AAATTTCCATGCCAAGAAGG | BF273477 |
| 12 | RM12FP  RM12RP | CCCGGTCTTCCAATAAGACT  GGATCACTGTCACCAAGCAT | BQ412362 |
| 13 | RM13FP  RM13RP | AAAGCCGATTCTCTTTGGAA  AGAGAAAGCCCATAGCCAGA | BG447461 |
| 14 | RM14FP  RM14RP | TCCGTACCTTGAAACCCTTC  TTGGAATAGATCCCGAGAGG | BG441980 |
| 15 | RM15FP  RM15RP | CTGGGTCGATCCCTGATATT  CACCAAGAACACAACCAATGA | BF269236 |
| 16 | RM16FP  RM16RP | CCGGGATTCTTGTTCACTTT  CGGGATTGATCCGTTTAACT | BF273652 |
| 17 | RM17FP  RM17RP | AAATGTGGGAAGGCTTGGT  AGCATGGAGATGGGTGATG | BQ407203 |
| 18 | RM18FP  RM18RP | TAAAGCAGGGAATGGAAAGG  CTTTGGCATTAAGTTTGGCA | BF271799 |
| 19 | RM19FP  RM19RP | AATTACTTGCGGGAAGAGGA  CAAGTAAGCCTTTGCCCAAT | BF273688 |
| 20 | RM20FP  RM20RP | AGCCCTTGATGTCCTTGACT  AACATGAGAATCGCAAGCAC | BQ411434 |
| 21 | RM21FP  RM21RP | GAACGAGGAAAGCGTAGACC  CGAATTTGGTTCATCACTCG | BG445718 |
| 22 | RM22FP  RM22RP | ATCATCCACAGGGATGTCAA  CGAGTAGGACAATCCCATAGC | BQ405546 |
| 23 | RM23FP  RM23RP | ACCTGTCGAGGAACTTGCTT  ATTCATGACCTGCAACTCCA | BG441233 |
| 24 | RM24FP  RM24RP | TCCGTACCTTGAAACCCTTC  TTGGAATAGATCCCGAGAGG | JG854980 |
| 25 | RM25FP  RM25RP | ACCTGTCGAGGAACTTGCTT  TTTGTCCGCACAACCTATTG | BQ403907 |
| 26 | RM26FP  RM26RP | TAAGCACACAACCGAGAAGC  CGCACTGCTCTTAGCCAGTA | BF276058 |
| 27 | RM27FP  RM27RP | TTGCGGGAACATCTTACTGA  AGTGGCAATGGGATGTTGTA | BQ407587 |
| 28 | RM28FP  RM28RP | GTTTGGCCGAGTTTAAGTCC  CAACACTTCGAGCGACATCT | BF269489 |
